# Bankruptcy is an inevitable fate of repeated investments with leverage

**DOI:** 10.1038/s41598-019-50237-6

**Published:** 2019-09-24

**Authors:** Momoka Nii, Takuya Okabe, Hiromu Ito, Satoru Morita, Yosuke Yasuda, Jin Yoshimura

**Affiliations:** 1 Graduate School of Integrated Science and Technology, Shizuoka University, Hamamatsu, 432-8561 Japan; 2 Department of International Health, Institute of Tropical Medicine, Nagasaki University, Nagasaki, 852-8523 Japan; 3 Department of Environmental Sciences, Zoology, University of Basel, Basel, 4051 Switzerland; 4 Department of Economics, Osaka University, Osaka, 560-0043 Japan; 5 Department of Environmental and Forest Biology, State University of New York College of Environmental Science and Forestry, Syracuse, NY13210 USA; 6 Marine Biosystems Research Center, Chiba University, Uchiura, Kamogawa, Chiba, 299-5502 Japan

**Keywords:** Applied mathematics, Acoustics

## Abstract

Due to the globalization and computerization of financial and economic activities, numerous repetitive leveraged investments have become possible in stock markets and currency exchanges. In reality, repeated leveraged investments up to 100 times/day are possible via online access. With computer-aided programs, this repetition number may easily increase 1000 times/day. The possibility of bankruptcy in repeated leveraged investments has never been considered in actual practices because the probability of bankruptcy in a single investment trial is almost negligible. Here, we show that the extremely numerous repetitions have a considerable chance of bankruptcy overall, even if the probability of bankruptcy for a single investment is extremely close to zero. The exact relationship between the repetitions and the probability of bankruptcy is approximated well by *n*(0.63) = *m*, where 10^*n*^ is the number of repetitions, 10^−*m*^ is the bankruptcy probability of a single investment, and *n*(0.63) is the 63% chance of bankruptcy. Thus, extremely rare events can always lead to bankruptcy in continuously repeated investment, even if the possibility of such an event is almost null. We suggest that the avoidance measure of bankruptcy is necessary in numerous repeated investments even if a single trial is almost certain to win.

## Introduction

Recent globalization has had a great impact on economic and financial activities in human society^1–11^. Until the 1980s, economic activities were mostly limited within regions, such as North America, Europe, and East Asia. Most personal investment activities were further limited to a single country because currency exchanges were also limited by high transaction fees^4,7,8,12,13^. Similarly, personal investments in stock markets were also highly limited due to high transaction fees for purchases and sales^1,7,13–15^. In the 1980s, these fixed transaction fees were lowered to almost negligible, and the transaction fees became mostly the quantity-based charge for frequent repeated investors^4,7,12,13^. These repeated investments are leveraged ten-twenty times larger than the transaction deposit. Thus the actual amount of investments (not the transaction deposit) may become larger than the total wealth of an investor. Therefore, the failure of an investment may cause bankruptcy because its debt may be larger than the whole wealth of the investor, although this probability is very small. Many financial funds (companies specializing in investment activities) had been established with the globalization of world economics since the 1980s^1,4,14^. The historical accounts and negative effects due to the changes in regulations are widely recognized in economics and business [for example, see^1,3,5,16–21^]. Thus, the basis of extremely numerous repeated investments with leverage became practical.

The recent developments in computer technology have had considerable impacts on the international economy^1,6^. The establishment of international computer networks had been introduced into stock markets and currency change. Together with globalization, the technical developments of computers and the establishment of international computer networks have induced important changes in economic and financial activities. Now, anybody can access any stock market or currency exchange by computer and make a transaction instantly from a distance, thus investment activities around the world being drastically increased.

Another facet of computerization is that stock market and currency exchange transactions can be completely automated by computer programing^14,22,23^. This automated investment makes it possible to repeat investments in a second interval. It is possible to make 60 transactions in a minute if the program simply states investments repeatedly. In practice, the program makes the investment decision (to send a transaction) when the price of a stock satisfies the condition set by the program. The total repetitions/hour may be ten or less. If the market is open for 10 hours, then the repeated investment occurs 100 times/day or 25,000 times/year. The maximum possible number of repetitions in investments is extremely high because of computerized investment. Established securities companies have shifted to adopt this computerized investment environment by hiring many specialist traders^24,25^. Many new investment funds or securities companies have also been established recently to specialize in this computerized global investment activity, e.g., long-term capital management (LTCM)^2,15,21^. The local operator-assisted environments of financial investments have thus shifted into the global computerized systems^1^.

In this paper, we consider a simple but forgotten feature of repeated investments. This aspect is mathematically trivial but has very profound effects on the investment strategy, once adopted. Traditionally, because the probability of bankruptcy for a one-time investment is nearly equal to zero, this one-time bankruptcy probability is usually treated as zero in current investment strategies. We here assume that this probability is very close to zero but not zero (a positive real number). We derive the mathematical relationship between the number of repetitions and overall bankruptcy in investments. We discuss the impact of this neglected aspect of repeated investments.

## Model and Results

In this repeated investment model, we suppose the situation in which the debts caused by leveraged investment may exceed the current wealth of the investor. The actual investments, e.g., FX investment, become at least ten to twenty times of transaction deposit by leverage and often easily surpass the whole asset (wealth)^26,27^, see also^28–30^. Assuming the number of repetitions of investment is *N*, the present wealth after *N* investments *w*_*N*_ is a function of the initial wealth *w*_0_ and the multiplicative growth rate *f*_*i*_ for *i* = 0, 1, …, (*N* − 1), such that ${w}_{N}={w}_{0}{f}_{0}{f}_{1}\ldots {f}_{(N-1)}$. Note that *f*_*i*_ is independent and random. We consider the case of *f*_*i*_ = 0, i.e., ${w}_{i+1}={w}_{i+2}=\ldots ={w}_{N}=0$. This means bankruptcy at the *i*-th investment. Let this probability be *p*_*b*_. In the presence of leveraged mechanisms, this value (*p*_*b*_) may be significantly small but still a positive number. Then, the total probability of bankruptcy with *N*-times repeated investments *P*_*B*_ is given by

1 $${P}_{B}=1-{(1-{p}_{b})}^{N}$$

**Figure 1:**
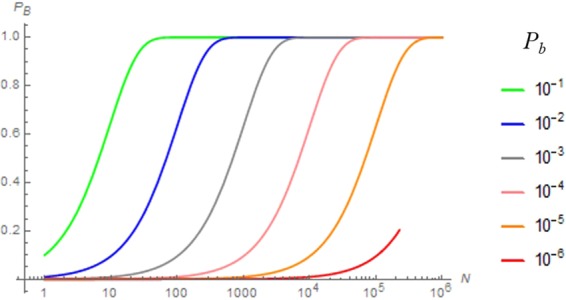
The relationship between the repetition times *N* (scaled on logarithm) and the overall probability of bankruptcy *P*_*B*_ for various rates *p*_*b*_ of bankruptcy in one investment trial. For any *p*_*b*_, *P*_*B*_ converges to 1 after a certain level of *N*.

By transforming Eq. (1), we obtain (Fig. 1):

2 $$N=\frac{\log (1-{P}_{B})}{\log (1-{p}_{b})}$$

**Figure 2:**
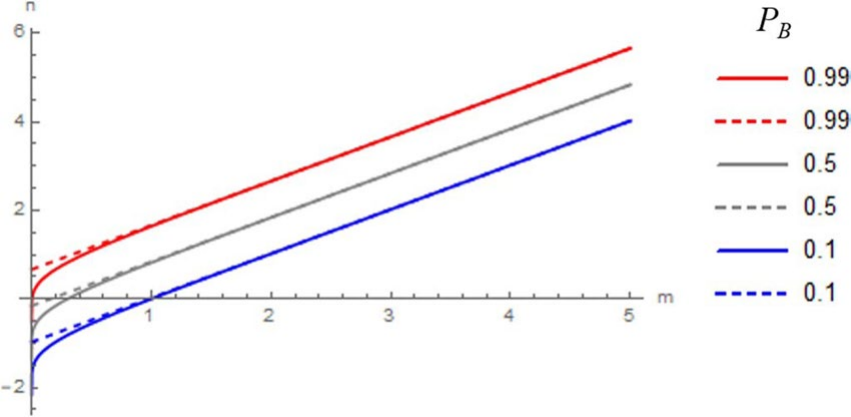
The relationship between the power *n* of repetition times and the negative power -*m* of one-time bankruptcy rate for a given overall probability of bankruptcy *P*_*B*_ (=0.1 (blue), 0.5 (grey) and 0.99 (red)) (solid lines: Eq. (3) and dashed lines: approximation by Eq. (5)).

Figure 1 shows that the overall probability of bankruptcy always converges to unity. It also shows that for a given *p*_*b*,_ there is a threshold of repetition *N*, where *P*_*B*_ reaches 0.99 or any given probability (Fig. 1). Expressing *p*_*b*_ = 10^−*m*^ and *N* = 10^*n*^, we obtain (Fig. 2):

3 $$n={\log }_{10}\frac{\log (1-{P}_{B})}{\log (1-{10}^{-m})}$$

Because *p*_*b*_ = ^−*m*^ is close to zero, *m*≫1. Using the first term of Taylor expansion, we can approximate $\log (1-{p}_{b})=\,\log (1-{10}^{-m})\cong -{10}^{-m}$. We obtain

4 $$n={\log }_{10}(\frac{\log (1-{P}_{B})}{-{10}^{-m}})=m+{\log }_{10}(\log \,\frac{1}{(1-{P}_{B})}).$$

Therefore, we can express (Fig. 2):

5 $$n=m+\alpha ({P}_{B}),$$

where $\alpha ({P}_{B})={\log }_{10}(\log \,\frac{1}{(1-{P}_{B})})$ is a measure of bankruptcy in repeated investments with leverage. For example, when *P*_*B*_ = 0.99, *α* = 0.66 (Fig. 2). Furthermore, Eq. (4) is approximated well by a linear relation (Fig. 2):

6 $$n=m+1.25{P}_{B}-0.79,\,{\rm{for}}\,0.3\le {P}_{B}\le 0.99$$

Therefore, we can obtain the following inequality:

7 $$n\ll m+1,\,{\rm{for}}\,\,{P}_{B}\le 0.99$$

Thus, when the one-time risk of bankruptcy is *p*_*b*_ = 10^−*m*^, we almost certainly face bankruptcy by repeating *N* = 10^*m*+1^ times. In precise, this repetition is approximately 99.995% bankruptcy.

In the case where the probability of bankruptcy, *p*_*bi*_, depends on each investment *i*, the total probability *P*_*B*_ is given by

8 $${P}_{B}=1-(1-{p}_{b1})(1-{p}_{b2})(1-{p}_{b3})\cdots .$$

Neglecting the second order terms in *p*_*bi*_, we obtain

9 $${P}_{B}\approx 1-{(1-\overline{{p}_{b}})}^{N},$$

where

10 $$\overline{{p}_{b}}=\frac{1}{N}({p}_{b1}+{p}_{b2}+\cdots ),$$

is the average of *p*_*bi*_. Therefore, this case is essentially the same as the above, except that *p*_*b*_ is replaced with $\overline{{p}_{b}}$. It is important to remark that *p*_*b*_ or $\overline{{p}_{b}}$ is very tiny but a positive number.

## Discussion

The current results indicate two important findings. First, any extremely small risk of bankruptcy can be accumulated to almost certainty (probability one) under repeated investments. Second, the number of repetitions leading to bankruptcy (*N* or *n*) is almost determined by the tiny probability of bankruptcy at each investment (*p*_*b*_ or *m*). For example, if the risk of bankruptcy is 10^−*m*^ (*m* is large), 10^*m*+1^ repetition almost certainly leads to bankruptcy (ca. 99.995%), and even 10^*m*^ repetitions have more than 50% bankruptcy (ca. 63.21%). This quantitative measure of bankruptcy *α* implies that computerized (programmed) repeated investment is a purely risky gamble, rather than a safe investment, even if a single trial is almost certain to win.

Globalization and computerization have made the current stock market and currency exchange systems qualitatively different from those of the 1970s or earlier. Traditionally, transactions (trading actions) in the stock market were made for long-term investment. Currency exchange was mostly for payments in international trading or international travellers. The fees for each transaction in these systems were a fixed constant that was so high, it was impossible to earn positive gains from a single short-term investment. After internet access to these trade systems became available, transaction fees lowered and varied depending on the amount of payments, so short-term repeated investments were able to earn positive gains. Then, traders had come to earn positive benefits from the repetition of short-term investments; while the one-time benefits were very small, numerous repetitions resulted in a large accumulated sum. Thus, the investment systems had moved from seeking large profits with slow returns to small profits with quick returns (SPQR) for both traders and securities companies receiving transaction fees.

The current market systems (both stock market and currency exchange) are occupied almost entirely by short-term investments with leverage. In stock markets, 95% or more buy-and-sell transactions are such estimated short-term investments, and traditional long-term investments are extremely rare, even though a large percentage of stocks are owned by long-term investors (e.g., parent companies and founder families) in superior enterprises. The situation for currency exchanges is similar to that for stock markets. Most transactions in currency exchanges are FX and other forms of short-term repeated investments with leverage (estimated to 95% or more, refs^22–24^). Payments for international trading and travellers, the primary purpose of currency exchanges, represent a very small portion of transactions in currency exchanges. Historically, the exchange rates of currencies were fixed (e.g., 360 yen/dollar) before 1973. Because of the 1973 oil crisis (the Nixon Doctrine), currency exchange moved from fixed rates to floating exchange rates^5^. At that time, a single transaction fee was fixed at a high rate, and it was impossible to earn profits by buy-sell repetitions in a short period. However, along with the development of computerized internet access, single transaction fees were adjusted so that FX and other repeated investments with leverage could produce benefits through numerous buy-sell actions using computer programs.

We should also note that globalization leads to the neutralization of the risk-spreading strategy^23,24^. In the previous decades, investment in many different companies was a very good portfolio strategy to avoid the risk of bankruptcy. Here, risk spreading works well with spreading (diversifying) the investment targets; this approach is the basic concept of portfolio selection developed by Harry Markowitz^1,31^. Due to economic globalization, all market movements or trends are highly correlated with each other. If one market is down, many others follow it. Thus, this high correlation between markets and companies makes risk spreading in leveraged repeated investments extremely difficult. Therefore, we need a new measure of bankruptcy avoidance for current repeated investments.

## Data Availability

No data are involved in this report.
